# Treatment of Complex Two-Vessel Coronary Heart Disease with Single Left Internal Mammary Artery as T-Graft with Itself—A Retrospective Double Center Analysis of Short-Term Outcomes

**DOI:** 10.3390/medicina58101415

**Published:** 2022-10-09

**Authors:** Christian Jörg Rustenbach, Ilija Djordjevic, Kaveh Eghbalzadeh, Hardy Baumbach, Stefanie Wendt, Medhat Radwan, Spiro Lukas Marinos, Migdat Mustafi, Mario Lescan, Rafal Berger, Christoph Salewski, Rodrigo Sandoval Boburg, Volker Steger, Attila Nemeth, Stefan Reichert, Thorsten Wahlers, Christian Schlensak

**Affiliations:** 1 Department of Thoracic and Cardiovascular Surgery, German Cardiac Competence Center, University of Tuebingen, 72076 Tübingen, Germany; 2 Department of Cardiothoracic Surgery, Heart Center, University of Cologne, 50937 Cologne, Germany; 3 Kardion GmbH, 70376 Stuttgart, Germany

**Keywords:** total arterial revascularization, LIMA, OPCAB

## Abstract

*Background and Objectives*: The strategy of revascularization may be constrained in patients with insufficient bypass grafts and with increased risk of wound healing disorders. Among those with complex left-sided double-vessel disease in whom a percutaneous coronary intervention (PCI), as well as the surgical procedure of minimally invasive coronary artery bypass grafting via left minithoracotomy (MICS CABG), is not a treatment option, CABG using the left internal mammary artery as a T-graft with itself may be an effective treatment strategy. *Materials and Methods*: We reviewed the data from patients treated in Cologne and Tuebingen from 2019 to 2022. We included 40 patients who received left internal mammary artery (LIMA) grafting, and additional T-graft with the LIMA itself. The objective was focused on intraoperative and short-term outcomes. *Results:* A total of 40 patients were treated with the LIMA-LIMA T-graft procedure with a Fowler score calculated at 20.1 ± 3.0. A total of 37.5% of all patients had lacking venous graft material due to prior vein stripping, and 21 patients presented severe vein varicosis. An overall of 2.6 ± 0.5 distal anastomoses (target vessels were left anterior descending, diagonal, intermediate branch, and/or left marginal ramus) were performed, partly sequentially. Mean flow of LIMA-Left anterior descending (LAD) anastomosis was 59.31 ± 11.04 mL/min with a mean PI of 1.21 ± 0.18. Mean flow of subsequent T-Graft accounted for 51.31 ± 3.81 mL/min with a mean PI of 1.39 ± 0.47. Median hospital stay was 6.2 (5.0; 7.5) days. No incidence of postoperative wound healing disorders was observed, and all patients were discharged. There was one 30-day readmission with a diagnosis of pericardial effusion (2.5%). There was no 30-day mortality within the cohort. *Conclusions:* Patients requiring surgical myocardial revascularization due to complex two-vessel coronary artery disease (CAD) can be easily managed with LIMA alone, despite an elevated Fowler score and a promising outcome. A prospective study needs to be conducted, as well as longer term surveillance, to substantiate and benchmark the long-term results, as well as the patency rates.

## 1. Introduction

The benefits of left internal mammary artery (LIMA) to left anterior descending coronary artery (LAD) grafting are well established and the selection of an additional graft to be used still remains fraught with controversy [1]. Nevertheless, total-arterial revascularization (TAR) is increasingly preferred due to its patency rates and long-term results. Even though, on the other hand, increased rates of deep sternal wound infections (DSWI), in particular subsequent to the use of the bilateral internal mammary artery (BIMA), emerge with substantial implications for morbidity, mortality, and additional costs for the treating institutions [2]. DSWI has been reported as requiring an extended course of antibiotics, an additional operation, or a combination of both. Coronary artery bypass patients at high risk of DSWI are increasing due to the gradual aging of the population, with more patients undergoing redo-surgery [3,4].

More co-morbidities, entailing both cardiovascular and infectious risks, are mainly diabetes mellitus and obesity in this patient group. Consequently, there is a growing urge to develop effective strategies for patients who are at high risk for DSWI preoperatively. The use of techniques, such as endoscopic vessel harvesting or skeletonized internal mammary artery, are used to reduce the risk of wound infection, whereas a simplified scoring system helps to assess an individual patient’s risk of suffering a severe infection after coronary artery bypass grafting (CABG) [5,6,7]. However, the operating team is often faced with the question of which strategy to follow when the radial artery or the great saphenous vein (SV) cannot be used in patients at high risk of DSWI. In a previously published study, we were able to report the first results regarding a new strategy to minimize this risk and also to achieve total-arterial myocardial revascularization [8].

**Figure 1:**
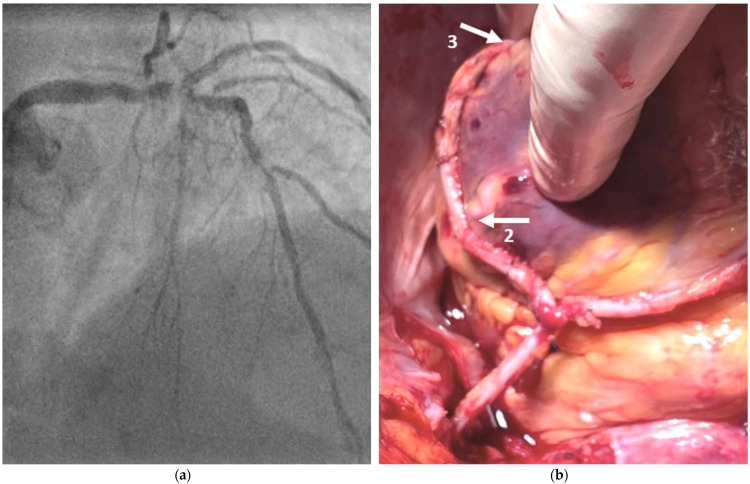
(**a**) Example image of a preoperative coronary angiography; (**b**) Result of intraoperatively performed revascularization.

We now outline our multicenter results and, in this context, discuss the presented option to use the left internal mammary artery (LIMA) on its own for multiple revascularizations of the ventricular anterior and lateral wall as a T-graft anastomosis with itself to provide a conceptual opportunity to perform total-arterial myocardial revascularization, even in the presence of insufficient graft material, and considerably decrease the risk of DSWI after CABG, particularly in complex two-vessel disease where interventional cardiology is not a viable treatment option (Figure 1). CABG remains the gold standard for patients with coronary artery disease, including those with diabetes and/or complex left main or three-vessel disease.

## 2. Materials and Methods

Owing to the retrospective design of the study, the Institutional Review Board of the University Hospitals of Cologne and Tübingen waived the need for informed consent. Between March 2019 and February 2022, 40 patients were admitted to our study. Descriptive statistics were performed to analyze the in-hospital outcomes for patients with left-sided double vessel disease, and multiple arterial grafting with a single left internal mammary artery as a T-graft (LIMA-LIMA T-graft) with itself in an isolated off-pump CABG (OPCAB) technique.

Among all of the patients evaluated for this specific surgical procedure, six were excluded from the analysis because either the corresponding angulation for the necessary anastomoses was excessive, the distance between the anastomoses was too far, or the LIMA could not be prepared for the required length. In three of these patients, after initial surgical treatment of at least the LAD and/or the diagonal branch, our cardiological team attempted to use a complex PCI procedure (e.g., rotablation) as a hybrid procedure to revascularize the remaining coronary vessels (e.g., marginal branch, intermediate branch), assuming a risk–benefit analysis necessitated to do so. In two patients, the lesser saphenous vein could be utilized. Regrettably, one patient was treated only partially successfully with interventional treatment (PCI).

### 2.1. Applied Criteria for LIMA-LIMA T-Graft

**Figure 2:**
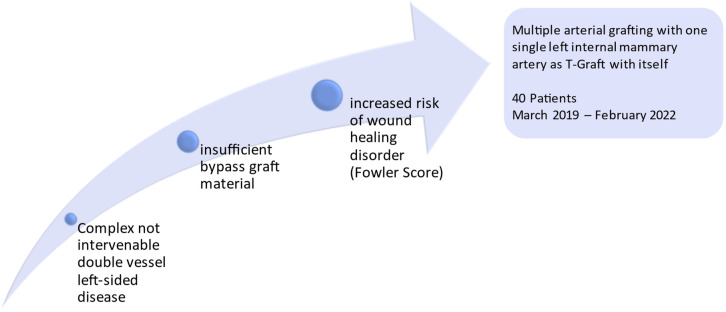
Criteria applied for LIMA-LIMA T-graft approach and flow chart of performed surgeries.

Eligibility criteria for a multiple LIMA-LIMA-T graft are illustrated in Figure 2. A Fowler score > 12 patients were not selected for a bilateral internal mammary artery (BIMA) graft because of an increased risk of DSWI. In addition, a LIMA-LIMA-T graft was chosen as the surgical revascularization strategy in patients with additional graft material limitation.

### 2.2. Surgical Technique

Full-length median sternotomy was used to perform the surgical procedure. The LIMA graft was accurately skeletonized at full length. Afterwards, in order to prove the feasibility of a double revascularization with LIMA and a T-graft, the LIMA was measured with a cord divided into two parts after the LIMA-LIMA T-graft was found to be feasible intraoperatively if the angle between the target vessels was significantly < 60° and the distance < 6 cm [9]. The objective and top priority of surgery was to achieve complete revascularization. Anastomoses were performed with 8-0 sutures (PROLENE^®^ Polypropylene Suture, Ethicon US, LLC. 2020. 085133-171129). In certain procedures, the anastomoses (e.g., LIMA on a diagonal branch, and, further, on a LAD or intermediate branch, and, further, on a marginal branch) were performed sequentially. Transit time flowmetry to assess bypass flow was routinely achieved by using a duplex ultrasonic probe (Medistim, Oslo, Norway).

### 2.3. Survey Objectives

Of the present study, the primary endpoint was in-hospital mortality. Secondary endpoints were serious adverse cardiac events (MACE) (defined as either coronary artery disease, stroke, peripheral heart disease, heart failure, or CVD-related mortality) occurring while in hospital [10].

### 2.4. Statistical Analysis

All statistical work was done using the Statistical Package for Social Sciences, version 27.0 (SPSS IBM, Chicago, IL, USA). We presented all data as continuous or categorical variables. Categorical data were both given as totals and percentages. Continuous data were examined for normality using the Kolmogorov–Smirnov test and expressed as mean ± standard deviation (SD) for normally distributed variables or median (interquartile range) for non-normally distributed continuous variables.

## 3. Results

The characteristics of pre-operative patients are shown in Table 1. Every one of the 40 patients were enrolled for the analysis. The average age of the predominantly male patients (*n* = 27, 67.5%) was 71.9 ± 7.1 years. The mean body mass index was 30.1 ± 2.3 kg/m^2^. Forty percent of the included patients (*n* = 10) had a history of coronary intervention with consecutive implantation of one or more stents. In addition, seven patients had a prior surgical myocardial revascularization. The syntax score [11,12] for the studied cohort was 32.1 ± 7.4. Regarding the applicable criteria for LIMA-LIMA-T grafts, 52.5% of the patients (*n* = 21) had severe varicosis. In 37.5%, it was not possible to use venous grafts because vein stripping had been performed previously. In a further 26 patients, radial artery harvesting was not feasible due to a positive Allen’s test. The mean Fowler score was 20.1 ± 3.0.

Table 2 summarizes the most relevant intraoperative data. A total of 57.5% (*n* = 23) of the surgical procedures that were performed were elective ones, 25% urgent and 17.5% emergent. The mean total revascularized coronary arteries was 2.6 ± 0.5, while the mean flow of the left internal mammary artery graft to left anterior descending artery (LIMA-LAD) was 59.31 ± 11.04 mL/min with a PI of 1.21 ± 0.18, whereas the mean flow of the LIMA-T graft was 41.31 ± 3.81 and 1.39 ± 0.47.

**Figure 3:**
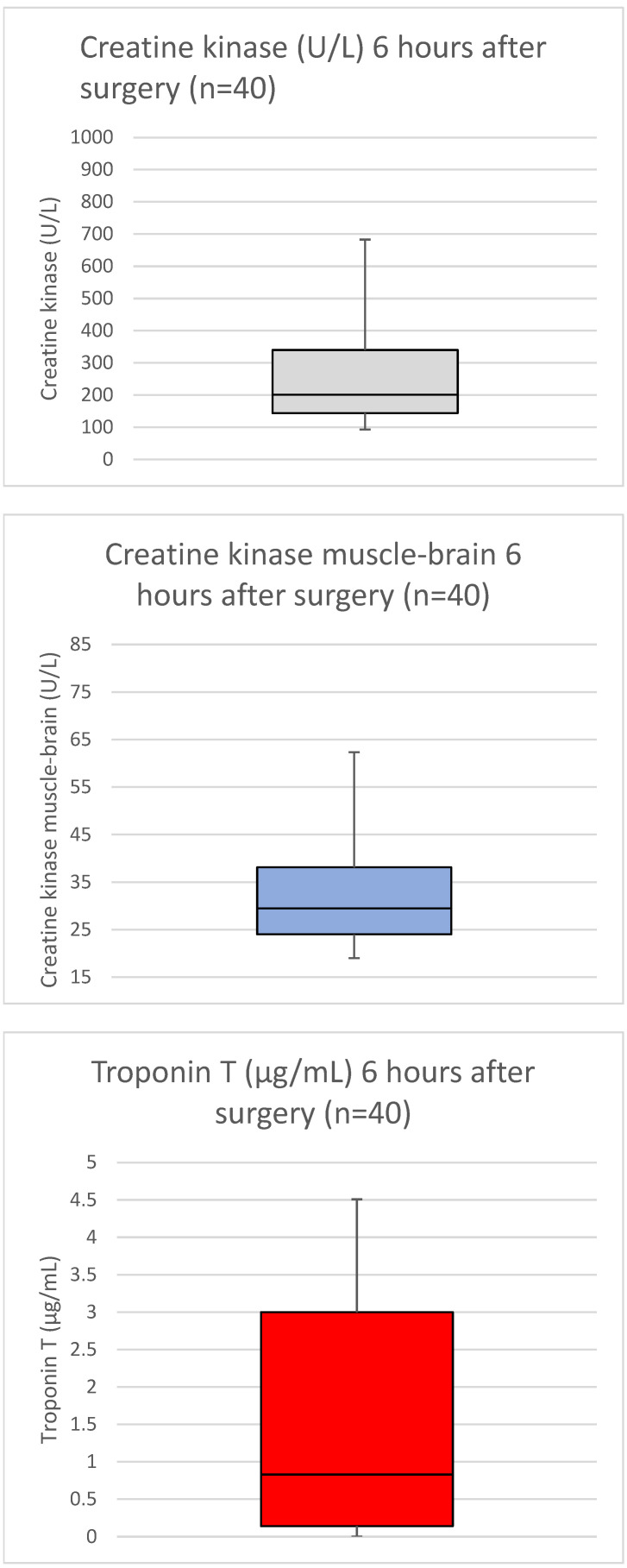
Boxplots presenting median and interquartile range of ischemia-related cardiac enzymes 6 h after performed surgery.

The postoperative cardiac enzymes (median creatine kinase 347.95 ± 298.69 U/L; median creatine kinase muscle-brain 34.25 ± 12.94; and median troponin T 1.26 ± 1.63 µg/L) suggested no evidence of acute myocardial ischemia (Figure 3).

Post-operative details are shown in Table 3. In the cohort analyzed, no re-thoracotomy and deep sternal wound infection occurred. One superficial wound healing disorder with no need of surgical treatment occurred (2.5%). No major adverse cardiac events were reported. Only two patients received a transfusion of red blood cell concentrates (5%). For all 40 patients (100%), discharge was after an average of 7 (6.6;7.2) days. No in-hospital death was observed.

To demonstrate the feasibility and safety of multiple arterial grafting using a single T-graft of the left internal mammary artery with itself for the treatment of complex two- or three-vessel diseases not amenable to interventional therapy in patients lacking bypass graft material and at increased risk of wound healing disorder was the purpose of this multicenter, retrospective study.

In this special cohort of patients, there was insufficient bypass graft material available, partly because the veins were not available due to stripping or varicose veins, and also because peripheral arterial occlusive disease was often present. Autologous grafts from the great saphenous vein (SVG) remain the most commonly used (80%) bypass conduits in coronary artery bypass grafting (CABG) despite the suggested benefits of multiple arterial grafts [13], which could not be used in these patients for the mentioned reasons. In addition, radial artery harvesting had been restricted due to the positive Allen´s test. Because the cohort of patients had an increased risk of wound healing disorders, BIMA was not evaluated as an optimal strategy choice for these patients.

BIMA was not the first-line therapy of choice for this particular patient cohort for the following reason: Regardless of recently published data suggesting that BIMA is not associated with a higher incidence of wound healing complications [14], the Arterial Revascularization Trial (ART), a key investigation focusing on this complication, has shown a nearly two-fold higher rate of sternal wound infections in the BIMA cohort (3.5% vs. 1.9%, *p* = 0.005) and a three-fold need for sternal wound reconstruction (2.0 vs. 0.6, *p* < 0.02) were shown [15]. A Fowler score of 20.1 ± 3.0 has already predict a significantly increased preoperative risk for sternal infection of more than 9.9% in our patient population [7].

As described previously, the possibility of revascularization with only the LIMA as a T-graft with itself was evaluated considering the quality and length of the graft, as well as the planned target vessels. Unless the IMA or a venous graft was available, however, harvesting for complete revascularization and patient safety was a viable option at all times. By using the off-pump procedure, the possibility of myocardial damage from prolonged graft harvest, if necessary, was essentially eliminated.

Sequential anastomosing of the coronary arteries with LIMA (except diagonal branch and left anterior descending) was not possible in all 40 included patients, as this would have resulted in incomplete revascularization. A previous study has described the importance of the angle and distance between the target vessels in order to have an effective T-graft anastomosis [9].

In 2019, Samadashvili et al. published an analysis of the New York database; patients who received multiple arterial grafts had significantly lower 7-year mortality compared with patients who received a single arterial graft (12.7% vs. 14.3%, *p* < 0.001) [16]. These data suggest that the survival of patients undergoing TAR is improved. Likewise, the superiority of arterial over venous revascularization has been demonstrated in other studies.

Analysis of the data available to us showed that all patients underwent complete revascularization, and one patient experienced mild wound healing disturbances with redness and wound secretion for 3 days, but subsequent reconvalescence. Our data indicate that a multiple arterial graft with a single LIMA and an additional T-graft with the LIMA itself for the treatment of complex left-sided double vessel disease is both feasible and safe. This could also relate to all procedures being performed by highly skilled OPCAB surgeons.

A prospective study design and a more substantial sample size would provide a more thorough evaluation of the described surgical approach to performing single-vessel TAR as initial treatment for high-risk patients for deep sternal wound infection. To prove the potential benefits of the LIMA-LIMA treatment, a long-term follow-up is mandatory. If proven noninferior or even superior, radial artery or great saphenous vein harvesting may be unnecessary in patients with left-sided double-vessel disease and concomitant high risk of deep sternal wound infection. Less invasiveness and fewer limb wound complications could contribute to better convalescence.

## 4. Study Limitations

There are some limitations to the presented study that need to be considered. As this is a retrospective study, the validity of the results depends on accurate and complete reporting within the dataset. Due to the small and exclusive patient cohort, the statistical power is quite moderate. Follow-up data exceeding the readmission results we presented were available; furthermore, the database is limited as it can only capture patients who were readmitted to the same hospitals. The study focused only on the feasibility and safety of the technique described, paying no special attention to long-term outcomes, and we did not have a control group with patients undergoing BIMA or single IMA with saphenous vein or radial artery for this special issue.

## 5. Conclusions

In conclusion, this study demonstrates once more that left internal mammary artery as T-graft with itself to treat complex left-sided double-vessel disease is feasible and safe in patients with missing bypass graft material and increased risk for deep sternal wound infection. There is still some need, nevertheless, to verify these results in long-term studies, and ideally, with a prospective study design to improve the generalizability and reproducibility of this technique.

## Figures and Tables

**Table 1:** Patient characteristics prior to surgery (*n* = 40).

Parameter	*n* = 40
Age—years	71.9 ± 7.1
Male sex—*n* (%)	27 (67.5)
Body-mass index	30.1 ± 2.3
Current smoker—*n* (%)	11 (27.5)
Ex-smoker—*n* (%)	24 (60.0)
**Co-morbidities**	
Hyperlipidemia—*n* (%)	40 (100)
Hypertension—*n* (%)	39 (97.5)
Pre-existent Stroke—*n* (%)	3 (7.5)
Chronic lung disease—*n* (%)	8 (44.5)
Immunosuppressive therapy—*n* (%)	3 (7.5)
Peripheral vascular disease—*n* (%)	19 (47.5)
Cerebrovascular disease—*n* (%)	6 (15.0)
Previous percutaneous coronary intervention—*n* (%)	18 (45.0)
Previous coronary artery bypass surgery—*n* (%)	7 (17.5)
Previous valve surgery—*n* (%)	2 (5.0)
Diabetes mellitus—*n* (%)	27 (67.5)
With HbA1c > 8.5—*n* (%)	7 (17.5)
Previous myocardial infarction—*n* (%)	6 (33.3)
Atrial fibrillation—*n* (%)	11 (27.5)
Dialysis—*n* (%)	0 (0)
Renal failure, without dialysis—*n* (%)	4 (10.0)
NYHA class IV—*n* (%)	3 (7.5)
Ejection fraction	46.1 ± 8.5
Cardiogenic shock—*n* (%)	0 (0)
Syntax – Score	32.1 ± 7.4
**LIMA-LIMA T-graft criteria**	
Varicosis—*n* (%)	21 (52.5)
Vein stripping—*n* (%)	15 (37.5)
Allen’s test positive—*n* (%)	26 (65.0)
Fowler score	20.1 ± 3.0

**Table 2:** Intraoperative data for the cohort analyzed (*n* = 40).

Parameter	*n* = 40
**Urgency of procedures**	
Elective—*n* (%)	23 (57.5)
Urgent—*n* (%)	10 (25.0)
Emergent—*n* (%)	7 (17.5)
**Operative data**	
Number of distal anastomoses	2.6 ± 0.5
Flow LIMA-LAD (mL/min)	59.31 ± 11.04
PI LIMA-LAD	1.21 ± 0.18
Flow T-graft (mL/min)	41.31 ± 3.81
PI T-graft	1.39 ± 0.47
Incision to closure time (min)	121.5 (106.7; 150)

PI, pulsatility index.

**Table 3:** Postoperative outcomes (*n* = 40).

Parameter	*n* = 40
**Postoperative complications**	
Deep sternal wound infection	0 (0%)
Wound healing disorder	1 (2.5%)
Re-thoracotomy	0 (0%)
Major adverse cardiac events	0 (0%)
**Outcomes**	
ICU stay (days)	1.0 ± 0.4
Total hospital stay (days)	6.6 (6.0; 7.2)
Discharged from hospital	40 (100%)
Intra-hospital mortality	0 (0%)

ICU, intensive care unit.

## Data Availability

The data presented in this study are available on request from the corresponding author. The data are not publicly available due to ethical and legal aspects.
